# Biouptake Responses of Trace Metals to Long-Term Irrigation with Diverse Wastewater in the Wheat Rhizosphere Microenvironment

**DOI:** 10.3390/ijerph16173218

**Published:** 2019-09-03

**Authors:** Renfei Li, Yuan Zhang, Hong Yu, Qiuling Dang, Hanxia Yu, Beidou Xi, Wenbing Tan

**Affiliations:** 1 State Key Laboratory of Environmental Criteria and Risk Assessment, Chinese Research Academy of Environmental Sciences, Beijing 100012, China (R.L.) (B.X.); 2 State Environmental Protection Key Laboratory of Simulation and Control of Groundwater Pollution, Chinese Research Academy of Environmental Sciences, Beijing 100012, China; 3 Institute of Geographical Sciences, Hebei Academy of Sciences, Shijiazhuang 050011, China; 4 College of Water Sciences, Beijing Normal University, Beijing 100875, China; 5 School of Life Sciences, South China Normal University, Guangzhou 510631, China

**Keywords:** soil rhizosphere microenvironment, trace metal speciation, soil physicochemical properties, bioavailability, wastewater irrigation

## Abstract

Wastewater irrigation is widely practiced and may cause serious environmental problems. However, current knowledge on the effects of long-term irrigation with wastewater from different sources on the biouptake of trace metals (TMs) in the rhizosphere zone by plants in farmlands is limited. Here, we analyzed wheat rhizosphere soil and wheat roots collected from a typical wastewater irrigation area in North China to evaluate the influence of wastewater irrigation from different sources on the bioavailability of trace metals in soils. Results showed that irrigation with tanning and domestic wastewater helped enhance the bioavailability of trace metals in rhizosphere soil by increasing the active organic carbon content, soil redox potential, and catalase activity, thus enhancing the proportion of the potentially bioavailable part of trace metal speciation. Conversely, irrigation with pharmaceutical wastewater can reduce the bioavailability of trace metals in rhizosphere soil by increasing total soil antibiotics and thus decreasing the proportions of bioavailable and potentially bioavailable parts of trace metal speciation. These findings can provide insights into the migration and transformation of trace metal speciation in soil rhizosphere microenvironments under the context of wastewater irrigation.

## 1. Introduction

Wastewater irrigation is a worldwide issue, and it is particularly common in developing countries [1,2]. This situation may become widespread in the future because of fresh water scarcity, population growth, urbanization, and increasing food demands [3]. Although wastewater irrigation can provide nutrients for soil development and boost agricultural productivity [4], it leads to the accumulation of soil contaminants, such as trace metals (TMs) and toxic chemicals [5], which may have considerably negative effects on the growth of crops and even threaten the health of humans and livestock through the food chain process [6,7,8,9]. In addition, numerous soil properties, such as soil pH, organic matter (OM) content, cation exchange capacity (CEC), soil redox potential, and enzyme activity, can be potentially affected by long-term wastewater irrigation [10,11,12,13]. Altered soil properties can further influence the transport and transformation of soil contaminants [14].

TM contamination has become a severe issue due to its wide reach and persistence in soil over long periods [15]. Numerous previous studies have focused on the impact of wastewater irrigation on the total concentrations of TMs in agricultural soils [5,16,17]. However, the dominant factor in determining the toxic effects, mobility, and bioaccumulation potential of TMs is their chemical speciation [18]. Determining the mobility and availability of TM speciation in soil is crucial in assessing the availability of TM to plants. Sequential extraction, an operationally defined procedure, is widely used to study the chemical speciation of TMs and evaluate the potential mobility and bioavailability of TMs in soil [19].

Rhizosphere refers to the region of soil that is in close contact and interacts with the plant root system within the soil. The microbial activity and chemical conditions of rhizosphere soil, which is a transition zone between soil and plant roots, are sensitive to changes in the external environment [20,21]. The transport and transformation of TM chemical speciation in rhizosphere soil can directly affect the uptake of TMs by plant roots. Therefore, long-term irrigation with wastewater from different sources may exert various effects on the physicochemical properties of rhizosphere soil. Such changes in physicochemical properties might lead to different transport and transformation pathways of TMs, potentially resulting in varying bioavailability levels of TMs in the soil rhizosphere microenvironment.

The main sources of wastewater used for irrigation include domestic and industrial wastewaters [22]. The storage and transfer of TMs in farmlands irrigated with these wastewater types have been intensively investigated [15,21,23,24,25]. However, to date, comparisons of the possible effects of long-term irrigation with wastewater from diverse sources on the transport and transformation of TMs, particularly in the soil rhizosphere microenvironment, are limited. Irrigation wastewater in different regions varies considerably depending on local industry. Thus, comparing the influences of the bioavailability of TMs by irrigation with wastewater from different sources can improve the current understanding of the major mechanisms through which wastewater irrigation affects the toxicity of TMs in soil. This task is also crucial in assessing the potential risks posed by wastewater irrigation in different regions.

In this study, we analyzed rhizosphere soil and wheat roots in farmlands irrigated with wastewater from domestic sewage treatment plants, chrome tanning, and pharmaceutical factories in a typical wastewater irrigation area in North China. This study aims to assess the impact of irrigation with wastewater from different sources on the bioavailability of TMs and physicochemical properties of rhizosphere soil.

## 2. Materials and Methods

### 2.1. Study Area and Sampling

**Figure 1:**
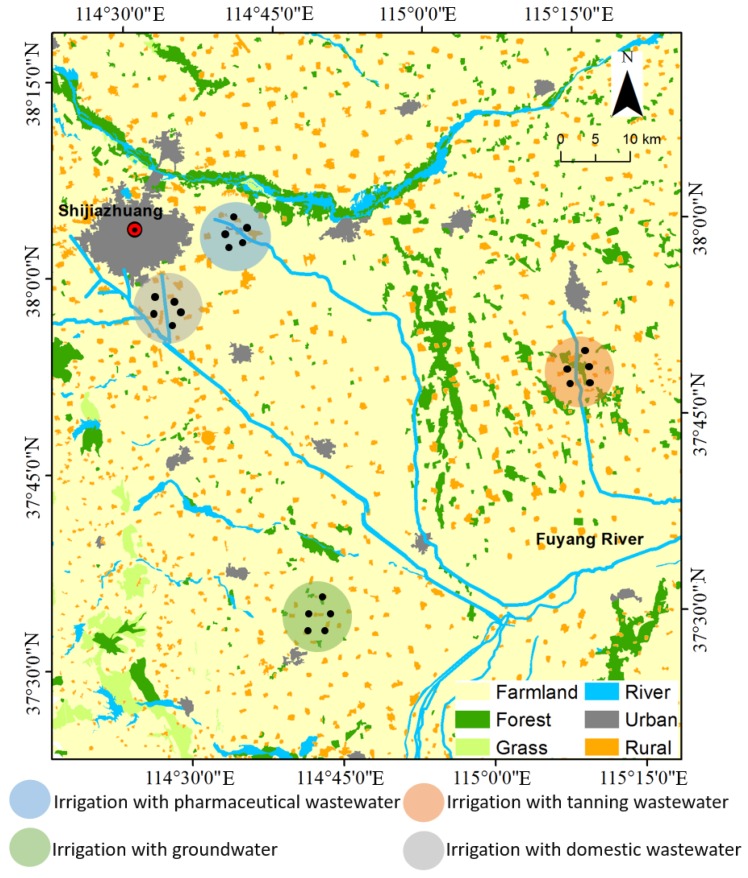
Map of the study area and sampling locations. Black dots in wastewater irrigation areas indicate sampling sites.

This research was conducted in a cereal crop-producing area of Shijiazhuang, the capital of North China’s Hebei province (Figure 1). The study area has a temperate continental climate with an annual average temperature of 11.5–13.9 °C, extreme maximum temperature of 40.5 °C, and extreme minimum temperature of −19 °C. The average annual precipitation is 600–650 mm (mainly experienced from June to August). The frost-free period covers 180–200 days. Summer corn–winter wheat is the main crop rotation pattern in this area and the typical soil type is Cambisol [26].

Shijiazhuang is one of the most important pharmaceutical and leather production bases in China. Certain farmlands in this area have been irrigated with wastewater for more than 30 years as a result of high demand for agricultural irrigation and the simultaneous existence of domestic sewage treatment plants, chrome tanning, and pharmaceutical factories [27]. Three typical wastewater irrigation farmlands, namely, tanning wastewater irrigated farmlands (TWIF), pharmaceutical wastewater irrigated farmland (PWIF), and domestic wastewater irrigated farmland (DWIF) were selected as research objects (Figure 1). A groundwater irrigated farmland was selected as the control. The total farmland area in Shijiazhuang for domestic wastewater irrigation, pharmaceutical wastewater irrigation and tanning wastewater irrigation are about 1246, 1880, and 1304 ha, respectively. The climatic conditions, soil type, crop type, fertilization level, and irrigation level of the farmlands have been consistent for a long time because these farmlands are located in the grain-producing areas of the North China Plain and are relatively close to one another.

Wastewater-irrigated and reference samples were collected in June 2014 (prior to harvest). Intact wheat roots were randomly sampled at five locations in each farmland. After transportation to the laboratory, rhizosphere soils were collected by using the adhering soil method. Briefly, after gently shaking the wheat roots by hand to remove bulk soils, the soil still adhering to the root surface (2 mm thick at the most) was considered rhizosphere soil. The rhizosphere soil was carefully brushed off from the roots, air-dried at room temperature, and sieved through a 2-mm mesh to remove soil fauna, fine roots, and rock fragments. After removing the rhizosphere soil, the roots were cleaned with deionized water and sealed in plastic bags. A total of 20 wheat root and 20 rhizosphere soil samples were stored at −20 °C for subsequent analysis. The effluents from the local sewage treatment plant, pharmaceutical plant, and tannery all met the integrated wastewater discharge standard of China (Table S1). Irrigation wastewaters and groundwater were collected in March, June, September, and November 2014, and their average physicochemical properties are presented in Table S2.

### 2.2. Soil Physicochemical Analysis

Soil pH was measured in a soil water suspension (soil–water ratio of 1:2.5). Soil redox potential (Eh) was measured with oxidation−reduction potential (ORP) depolarization automatic analyzer (FJA-6, Nanjing Chuan-Di Instrument & Equipment, Nanjing, China). Soil clay content (<2 μm) was determined using a laser particle size analyzer (Malvern Mastersizer, 2000, Malvern, UK), and the cation exchange capacity (CEC) was determined using standard methods [28]. The concentrations of N and S were determined with an elemental analyzer (Vario EL cube). The total organic carbon (TOC) and soil dissolved organic carbon (DOC) extracted with 0.01 M CaCl_2_ (soil: solution ratio of 1:10; 2 h) were determined with a Shimadzu 5000 TOC analyzer. Permanganate oxidizable carbon (KMnO_4_-C) was determined using the method of Vieira et al. [29]. Soil antibiotics were extracted according to the modified method of Xie et al. [30], and the target antibiotic compounds included chlortetracycline, cefazolin, cefotaxime, cefoxitin, cefaclor, cefuroxime, furazolidone, sulfadiazine, sulfamerazine, sulfamethazine, sarafloxacin, oxytetracycline, ofloxacin, trimethoprim, tetracycline, and carbadox. For the concentrations of P, K, Ca, Fe, Mn, and Mg, soil was extracted by aqua regia at 160 °C, and the concentrations were determined through inductively coupled plasma-optical emission spectrometry (ICP-OES, Thermo ICAP-6000, Waltham, MA, USA).

### 2.3. Soil Biomass Carbon and Enzyme Activity Determination

Aliquots of the fresh rhizosphere soil samples were used to determine microbial biomass carbon (MBC) via a modified fumigation extraction procedure [31]. The activity of soil catalase was measured using the method of Zhou et al. [32]. Briefly, 3% H_2_O_2_ as the oxidizer was added to fresh soil and allowed to stand for 30 min at 3 °C. Afterward, the reaction was stopped with the addition of 1 M H_2_SO_4_. After filtration, 0.5 M H_2_SO_4_ was added to the filtrate, and 20 mM KMnO_4_ was used to measure the O_2_ absorbed. The activities of lignin peroxidase (LiP), laccase (Lac), and manganese peroxidase (MnP) were assayed using the methods of Fujii et al. [33]. The MBC and enzyme activities of rhizosphere soils irrigated with groundwater and different wastewater types are presented in Table 1.

### 2.4. TM Analysis

The chemical speciation of TMs (Cu, Cr, Cd, As, Pb, and Ni) in rhizosphere soils was analyzed with a modified five-step extraction method [34]. In accordance with Tessier’s method, metals were partitioned into the following five operationally defined fractions:

(1) Exchangeable fraction. Samples were extracted at a solid-to-solution ratio of 1:8 with 0.5 M MgCl_2_ (pH 7.0) and stirred continuously for 5 h.

(2) Fraction bound to carbonates. The residual from (1) was extracted with 1 M NaOAc (pH 5.0) at a solid-to-solution ratio of 1:8 and stirred continuously for 5 h.

(3) Fraction bound to Fe–Mn oxides. The residual from (2) was extracted with 0.04 M NH_2_·OH·HCl in 25% (v/v) HOAc (pH 2.0, solid-to-solution ratio of 1:20) and occasionally stirred for 6 h at 96 °C.

(4) Fraction bound to OM. The residue from (3) was extracted with 30% H_2_O_2_ (pH 2.0, solid-to-solution ratio of 1:20) and occasionally stirred for 6 h at 85 °C then extracted with 3.2 M NH_4_OAc in 20% (v/v) HNO_3_ and continuously stirred for 30 min.

(5) Residual fraction. The residue from (4) was digested with concentrated HNO_3_ and HClO_4_ acids at 90–190 °C for 18 h.

To further explore the transformation of TM chemical speciation in rhizosphere soil, water-soluble fraction and humic acid fractions were added to the steps shown above. Therefore, TMs in rhizosphere soils were extracted as the following seven fractions: fraction 1 (water soluble), fraction 2 (ion exchangeable), fraction 3 (bound to carbonates), fraction 4 (bound to humic acids), fraction 5 (bound to Fe–Mn oxides), fraction 6 (bound to OM), and fraction 7 (residual).

After microwave digestion, the total concentrations of TMs contained in wheat roots and the concentration of each chemical speciation of TMs in rhizosphere soils were analyzed through ICP-OES (Thermo ICAP-6000, Waltham, MA, USA).

### 2.5. Statistical Analysis

The bio-accumulation factor (BAF) is the ratio of TM concentration in wheat root to that in rhizosphere soil, and was calculated in this study as follows:

BAF = C_root_/C_soil_ (1)

where C_root_ and C_soil_ represent the TM concentration in wheat root and rhizosphere soil, respectively.

Data were expressed as arithmetic mean ± standard deviation calculated from replicates. The relationships between BAF and environmental variables, between TM chemical speciation and environmental variables (Figures S1–S3), and between environmental variables (Figures S4–S6) were assessed by correlation analysis. Analysis of variance (ANOVA) was performed to compare the differences in BAF values, physicochemical characteristics, and total concentration of TMs among farmlands irrigated with wastewaters from different sources. All data were subjected to homogeneity testing before ANOVA to ensure they were in a normal distribution. Stepwise multiple regression was used to evaluate the chemical speciation of TMs responsible for the changes in BAF values in different farmlands. Correlation analysis, stepwise multiple regression, and ANOVA were conducted with SPSS 20.0 (IBM Corporation Software Group, Somers, New York, NY, USA). The results were considered significant at the *p* < 0.05 level.

Structural equation models (SEMs) were constructed to determine the major pathways that affect the BAF of TMs in rhizosphere soils irrigated with different wastewater types. SEM is a modeling tool that integrates ANOVA, regression analysis, path analysis, and factor analysis to deal with multivariate complex relationships. SEM can be used to analyze the relationship between latent variables and simulate the internal logic of multiple factors [35]. The model χ^2^ test (*p* > 0.05), normed fit index (NFI > 0.90), goodness-of-fit index (GFI > 0.90), and low root mean square errors of approximation (RMSEA < 0.05) were used to indicate the overall fitness of SEMs. SEM analyses were performed using SPSS 20.0 and AMOS 21.0 (IBM Corporation Software Group, Somers, New York, NY, USA).

## 3. Results

### 3.1. Changes in Rhizosphere Soil Properties

The descriptive statistics of several physicochemical characteristics of the analyzed rhizosphere soils are summarized in Table 1. The TOC, DOC, and KMnO_4_-C contents in rhizosphere soils irrigated with wastewater increased in comparison with the control, possibly due to the high concentrations of OMs in wastewater (Table S2). The total soil antibiotic content in PWIF was significantly higher than that in other farmlands. Compared with the control, soil pH was higher in PWIF and had an average of 8.13. In contrast, lower soil pH was detected in TWIF and DWIF and had average values of 7.52 and 6.85, respectively. Soil Eh was lower with pharmaceutical wastewater irrigation (63.96 mV) but higher with domestic and tanning wastewater irrigation (85.34 and 95.98 mV, respectively) compared with the control. The mean concentrations of N, P, K, Ca, Mg, S, and Mn showed no significant differences in the farmlands irrigated with groundwater and wastewater. Compared with the control, the soil clay contents was higher in PWIF and had an average value of 39.5%, but it was lower in TWIF and DWIF and had average values of 34.47% and 30.64%, respectively.

Soil catalase activity, which is often related to the quantity and activity of aerobic microorganisms [36], increased with wastewater irrigation (Table 1). However, the soil biomass carbon decreased after pharmaceutical and tanning wastewater irrigation, which could be ascribed to the high levels of total antibiotics and toxic materials in pharmaceutical and tanning wastewater (Table S2). Similarly, the activity of LiP and Lac decreased with long-term pharmaceutical wastewater irrigation. Except for MnP, all of the analyzed enzyme activities increased with domestic wastewater irrigation.

### 3.2. TM Contents in Rhizosphere Soils

Table 2 summarizes the total concentrations of TMs in rhizosphere soils irrigated with groundwater and wastewater from different sources. China’s soil environmental quality standards are recommended by the Ministry of Environmental Protection of the People’s Republic of China (MEPPRC) [37]. These standards consist of three grades of threshold values, in which the first grade is the background values of the soil, the second grade is for pollution assessment in agricultural soils, and the third grade is for pollution assessment in forests or highly contaminated agricultural soils. The threshold of the second grade was used to evaluate the TMs in rhizosphere soils in this study.

Compared with the control, the concentrations of Cu, Cr, Cd, Pb, and Ni increased significantly after long-term tanning wastewater irrigation. Similarly, but to a reduced extent, the concentration of Cu increased remarkably after pharmaceutical wastewater irrigation, and the concentrations of Cu and Cr increased significantly after domestic wastewater irrigation compared with the control. Furthermore, in TWIF, the mean concentrations of Cu, Cr, Cd, and Pb exceeded the reference background values of Hebei province, but remained below the second grade of environmental quality standards. In PWIF and DWIF, the mean concentrations of Cr and Cd exceeded the reference background values, but remained below the second grade of standards. The total concentration of Ni exceeded the second grade of the environmental quality standard in both wastewater and groundwater irrigated croplands.

### 3.3. Distribution of TM Fractions in Rhizosphere Soils

Seven types of chemical speciation of TMs in rhizosphere soils were successfully extracted by sequential extraction and summarized in three parts according to their bioavailability, namely, a bioavailable (B) part, a potentially bioavailable (PB) part, and a non-bioavailable (NB) part. The bioavailable part represents the fractions likely to be directly absorbed by plants, and consists of fractions 1–3. The potentially bioavailable part can be absorbed by plants in strong acid medium and consists of fractions 4–6 [18]. The non-bioavailable (NB) part is residual fraction (fraction 7) and cannot be absorbed by plants [38].

**Figure 2:**
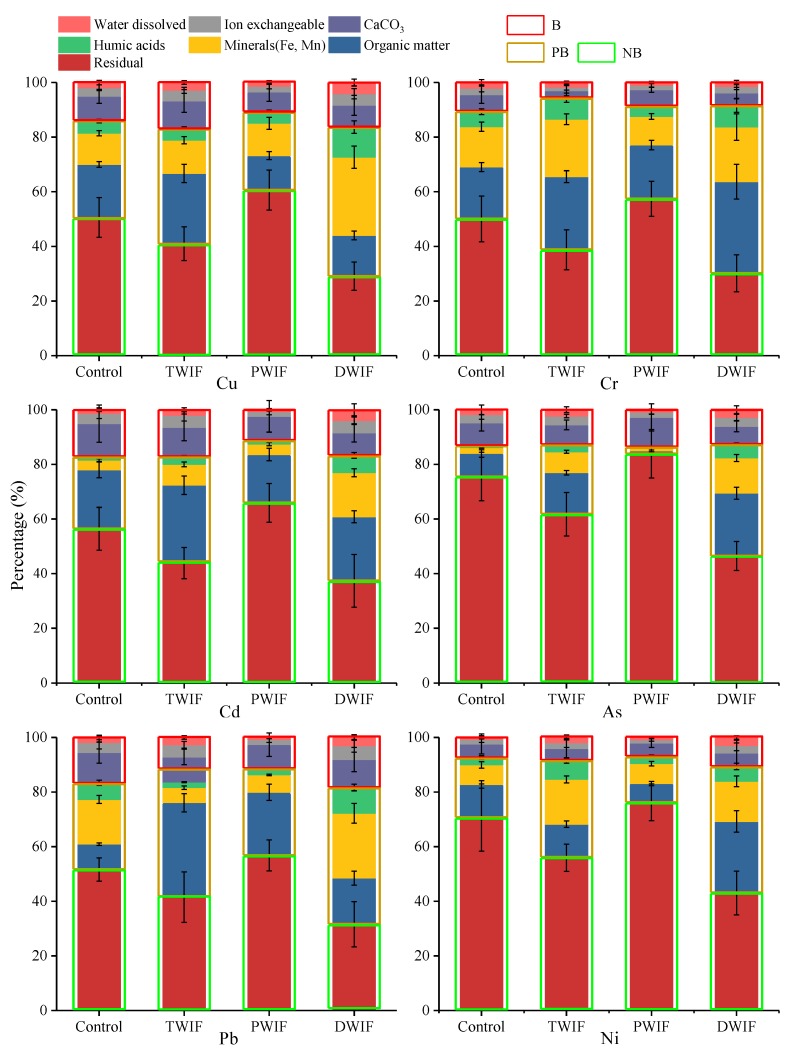
The proportions of TM chemical speciation in rhizosphere soils irrigated with groundwater and wastewaters from different sources. TWIF, PWIF, DWIF, and Control denote irrigation with tanning, pharmaceutical, domestic wastewater, and groundwater, respectively. B: bioavailable part; PB: potentially bioavailable part; NB: non-bioavailable part.

The proportions of TM speciation in rhizosphere soils irrigated with groundwater and different wastewater types are presented in Figure 2. The PB and B parts of the analyzed TMs were significantly higher in TWIF and DWIF and remarkably lower in PWIF than those in the control. On the contrary, the NB part of all TMs was notably lower in TWIF and DWIF, and significantly higher in PWIF than that in the control. These results show that pharmaceutical wastewater irrigation could increase the proportions of TMs in the NB part and decrease the proportions of TMs in the PB and B parts. By contrast, domestic and tanning wastewater irrigation could reduce the proportions of TMs in the NB part and increase the proportions of metals in the PB and B parts. Therefore, the bioavailability of TMs could be increased in rhizosphere soils irrigated with domestic and tanning wastewater and decreased in rhizosphere soils irrigated with pharmaceutical wastewater.

### 3.4. Differences in BAF among Farmlands Irrigated with Wastewater from Different Sources

**Figure 3:**
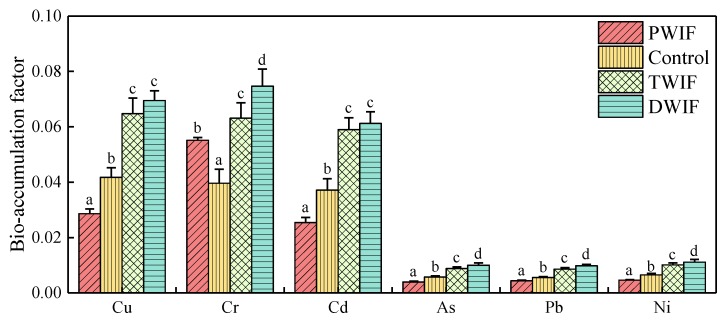
Bio-accumulation factors of each TM in rhizosphere soils irrigated with groundwater and wastewater from different sources.

BAF can be used to illustrate the strength of the enrichment of TMs by plant roots and was calculated according to the Equation (1). The bioaccumulation of TMs was observed in all tested wheat roots, as shown in Figure 3. Compared with the BAF values of the control, those of the analyzed TMs increased after domestic and tanning wastewater irrigation. Inversely, the BAF values of TMs (except for Cr) decreased with pharmaceutical wastewater irrigation. These results further confirm that irrigation with domestic and tanning wastewater could increase the bioavailability of TMs, whereas irrigation with pharmaceutical wastewater could decrease the bioavailability of TMs in the soil rhizosphere microenvironment.

### 3.5. Effects of Physicochemical Properties and TM Fractions on BAF in Farmlands Irrigated with Wastewater from Different Sources

After integrating the data of each wastewater-irrigated soil with the data of groundwater-irrigated soil (10 data pairs in total for each comparison), correlation analysis, stepwise regression analysis, and structural equation modeling were performed to analyze the relationships among BAF values, soil properties, and soil TM chemical speciation in different wastewater-irrigated rhizosphere soils.

**Figure 4:**
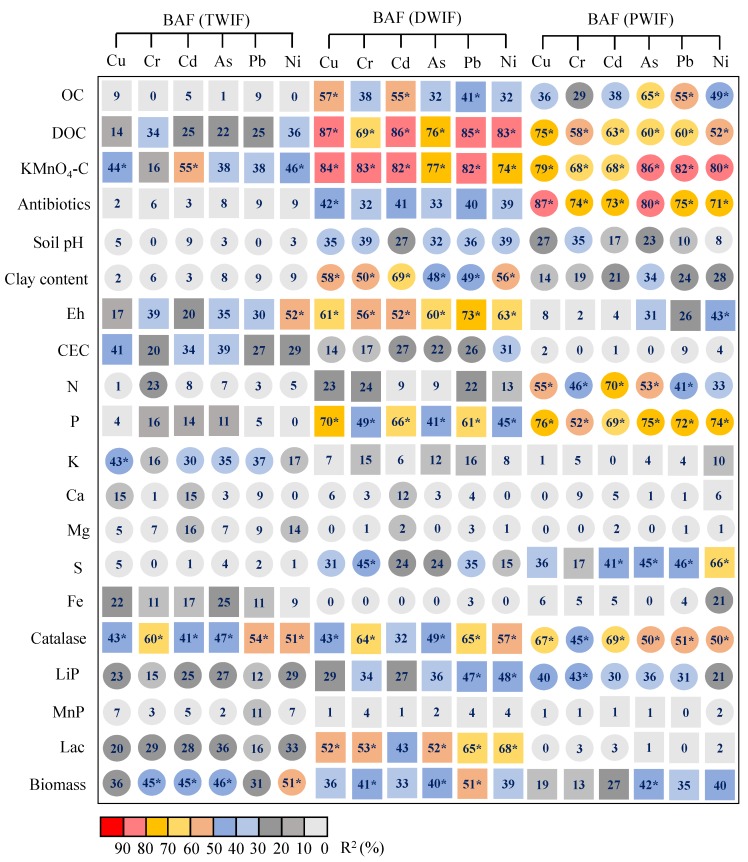
Correlation coefficients (R^2^) of the BAF values and indices of physicochemical properties in rhizosphere soils irrigated with wastewaters from different sources. TWIF, PWIF, and DWIF denote irrigation with tanning, pharmaceutical, and domestic wastewater, respectively. Squares and circles indicate positive and negative correlations, respectively. Significance of the correlations (*) are evaluated at the 0.05 levels.

The transformation and biouptake processes of TMs in soils are generally regulated by many physicochemical and biological properties. Thus, some soil physicochemical and biological indicators were selected and analyzed to investigate their influences on the BAF of TMs. The correlations between BAF values of TMs and soil properties showed evident differences among the farmlands irrigated with diverse wastewater types (Figure 4). For DWIF, the BAF values of TMs exhibited significantly positive correlations with DOC, KMnO_4_-C, P, catalase activity, and lac activity, and a remarkably negative correlation with clay content. For TWIF, the BAF values of TMs exhibited positive correlations with KMnO_4_-C content, Eh, and CEC, and a negative correlation with soil biomass carbon. For PWIF, the BAF values of TMs showed significantly negative correlations with DOC, KMnO_4_-C content, total antibiotics, clay content, N, P, and catalase, and a positive correlation with S.

**Figure 5:**
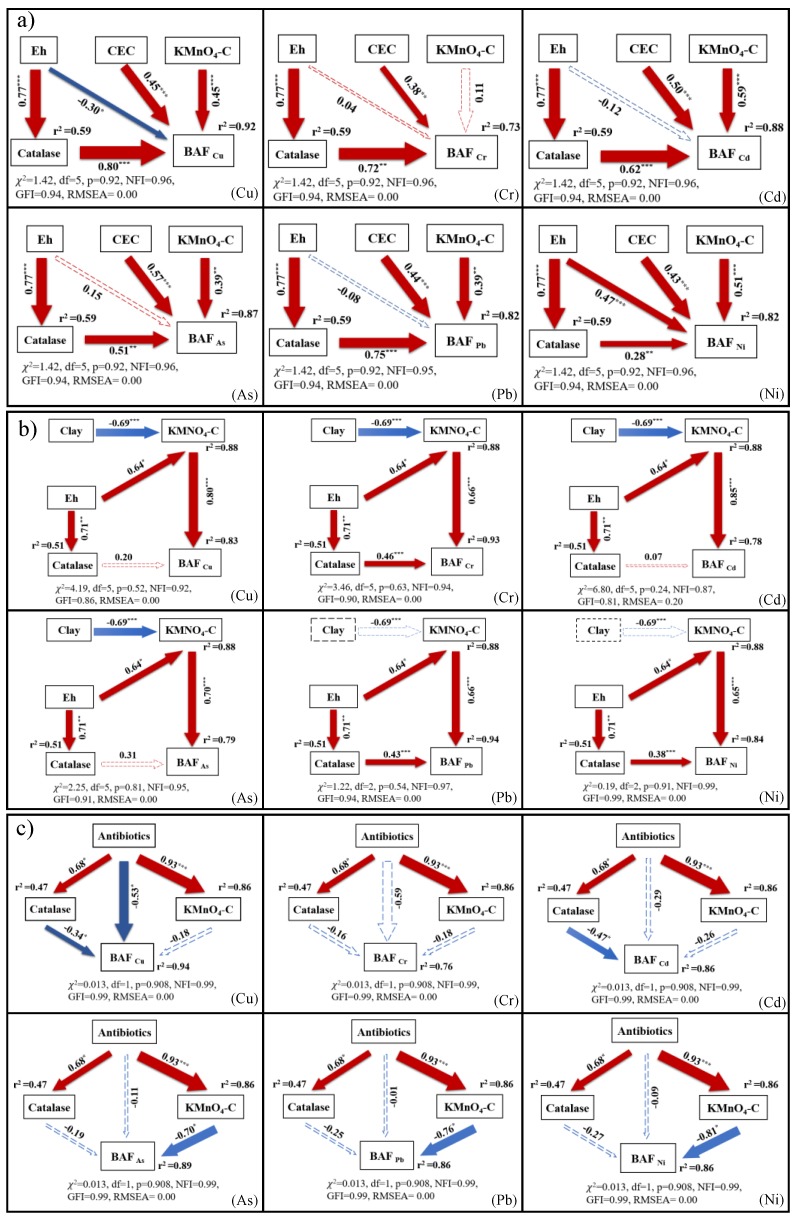
Results of the SEMs. Square boxes indicate variables, arrows connecting the boxes indicate the direction of causation. Red and blue arrows indicate positive and negative relationships, respectively. The arrow widths are proportional to the *p* values, which reflect the importance of the factors. *r*^2^ is shown near each response variable in the models, which represent the proportion of explained variance. The final model fit was evaluated by a χ^2^ test, normed fit index (NFI), goodness-of-fit index (GFI) and root mean square errors of approximation (RMSEA). * *p* < 0.05, ** *p* < 0.01, *** *p* < 0.001. (**a**) Irrigation with tanning wastewater. (**b**) Irrigation with domestic wastewater. (**c**) Irrigation with pharmaceutical wastewater.

After finding out some physicochemical and biological indicators that are strongly correlated with BAF, SEM was conducted to analyze the hypothetical pathways that may explain how these physicochemical and biological indicators affect BAF under different wastewater irrigation conditions. The evaluation parameters show that the SEMs constructed in this study were well-fitted (TWIF: χ^2^ = 1.42, df = 5, *p* = 0.92, NFI = 0.96, GFI = 0.94, RMSEA = 0.00, mean; DWIF: χ^2^ = 3.02, df = 4, *p* = 0.61, NFI = 0.94, GFI = 0.90, RMSEA = 0.03, mean; PWIF: χ^2^ = 0.013, df = 1, *p* = 0.91, NFI = 0.99, GFI = 0.99, RMSEA = 0.00, mean; Figure 5). On the average, our model explained 84% (Figure 5a), 85% (Figure 5b), and 86% (Figure 5c) of the variance in BAF values of TMs in rhizosphere soils irrigated with tanning, domestic, and pharmaceutical wastewaters, respectively. Among the explanatory variables, KMnO_4_-C content and catalase activity directly affected the BAF values of TMs in all wastewater-irrigated farmlands. For TWIF (Figure 5a), CEC, KMnO_4_-C content, and catalase activity had direct positive effects on the BAF values of all TMs, whereas the catalase activity was affected by soil Eh. For DWIF (Figure 5b), KMnO_4_-C content and catalase activity had direct positive effects on the BAF values of all TMs, soil Eh had direct positive effects on KMnO_4_-C content and catalase activity, and clay contents had a direct negative effect on KMnO_4_-C content. However, for PWIF (Figure 5c), KMnO_4_-C content and catalase activity had direct negative effects on the BAF values of all TMs, and total soil antibiotic content had a remarkably positive effect on KMnO_4_-C content and catalase activity.

**Figure 6:**
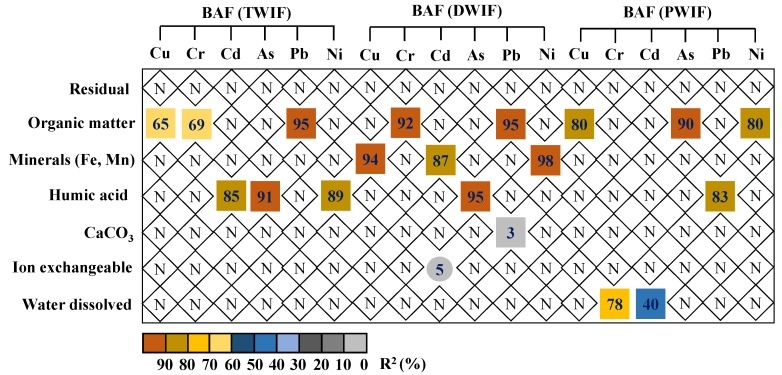
Stepwise multiple linear regression reflecting the changes in TM fractions on the BAF values in rhizosphere soil irrigated with wastewaters from different sources. Squares and circles indicate positive and negative correlations, respectively. Diamonds indicate statistically nonsignificant correlation.

The distribution of TM chemical speciation in soils greatly affects the biouptake processes of TMs. Since wastewater irrigations remarkably changed the distribution of TM chemical speciation in soils (Figure 2), stepwise regression analysis was conducted to illustrate the influences of these changes on the BAF values of TMs. The results indicate that the fractions bound to OM, Fe–Mn oxides, and humic acids were the dominant chemical speciation responsible for the variations in BAF values in the farmlands irrigated with wastewater from different sources (Figure 6). For the farmland irrigated with tanning wastewater, OM-bound fractions explained 65%, 69%, and 95% of the variations in BAF values of Cu, Cr, and Pb, respectively, while humic acid-bound fractions explained 85%, 91% and 89% of the variations in the BAF values of Cd, As, and Ni, respectively. For the farmland irrigated with domestic wastewater, the role of OM-bound and humic acid-bound fractions was replaced by Fe–Mn oxide fractions, which explained 94%, 87%, and 98% of the variations in the BAF values of Cu, Cd, and Ni, respectively. For the farmland irrigated with pharmaceutical wastewater, OM-bound fractions explained 80%, 90%, and 80% of the variations in the BAF values of Cu, As, and Ni, respectively, while humic acid-bound fractions explained 83% of the variations in the BAF values of Pb. Meanwhile, water dissolved fractions explained 78% and 40% of the variations in the BAF values of Cr and Cd, respectively.

## 4. Discussion

The bioavailability of TMs in rhizosphere soil exhibited significant differences in the farmlands irrigated with wastewater from different sources. Tanning and domestic wastewater irrigation led to an increase in the bioavailability of TMs, whereas pharmaceutical wastewater irrigation led to a decrease in the bioavailability of TMs (Figure 3). Our results also showed that changes in the PB part (humic acid- and OM-bound fractions) and B part (water dissolved fractions) of TM speciation were the determinants that resulted in the decrease in BAF values in PWIF, and changes in the PB part were the determinant that led to the increase in BAF values in TWIF and DWIF (Figure 6).

Furthermore, long-term irrigation with wastewater from different sources could lead to changes in several soil properties (Table 1). Decreased soil pH was observed in the rhizosphere soils with domestic wastewater irrigation (Table 1). We found that soil pH had an observably negative correlation with soil enzymatic activity in DWIF (Figure S5). However, a previous study reported that soil pH was positively correlated with soil enzymatic activities after wastewater irrigation [39]. The contradiction of both studies implies that there might be no direct causal relationships between the changes of soil enzymatic activities and soil pH in DWIF. Increased soil enzymatic activities and decreased soil pH in DWIF might be attributed to the additional input of active organic matter and exchangeable cations created by the irrigation water [40]. In PWIF, soil pH was elevated with an increase in soil OM content (Table 1, Figure S6). Mancino and Pepper [41] attributed this increase in soil pH after wastewater irrigation to an increase in denitrification rate. Thus, the denitrification rate might be strengthened in rhizosphere soil after long-term irrigation with pharmaceutical wastewater. Notably, antibiotic content was the most obvious difference between PWIF and other farmlands irrigated with wastewater (Table 1). Total soil antibiotic content was a key factor that affected the BAF values of TMs in PWIF (Figure 5). Accumulation of antibiotics could inhibit the target microorganisms, and other uninhibited microorganisms could acquire abundant resources to rapidly reproduce, thereby altering the compositions and functions of microbial communities in the soil [42]. Accordingly, the population of certain microbial species might be enhanced in PWIF due to the environmental selection of strains resistant to the antibiotics. Such changes may eventually lead to variations in some soil properties in PWIF, such as soil pH (Table 1) [41,43].

KMnO_4_-C content was one of the direct factors responsible for the variations in BAF values in the investigated rhizosphere soils (Figure 5). The close association between BAF values and KMnO_4_-C content was possibly due to OM, especially active-soil OM (DOC, KMnO_4_-C), which could regulate BAF values by affecting dissolved TM concentrations in soil [44]. KMnO_4_-C content exerted a positive effect on BAF values in TWIF and DWIF (Figure 5a,b). One possible explanation is that the accumulation of KMnO_4_-C content could lower soil pH (Table 1) [45], thus indirectly leading to a higher solubility of TMs and root uptake of TMs. However, in PWIF, KMnO_4_-C content exerted a negative effect on the BAF values of TMs (Figure 4 and Figure 5), suggesting that the major effect of KMnO_4_-C content on the bio-availability of TMs depends on the type of irrigation water. Generally, soil organic matter has a high affinity for metal cations due to the presence of ligands or functional groups such as carboxylic acids (-COOH), hydroxylic acids (-OH), and phenolic acids (aromatic ring-OH) [46]. Therefore, the negative effect of KMnO_4_-C content on BAF might be attributed to the fact that the accumulation of KMnO_4_-C content can directly lower the mobility and solubility of TMs in soils via adsorption and complexation processes. Moreover, the positive and negative effects of KMnO_4_-C content on the bio-availability of TMs might simultaneous exist during wastewater irrigation, and future studies need to explore the mechanisms regulating the major effects of KMnO_4_-C content on the bio-availability of TMs during wastewater irrigation.

Our results also revealed that catalase activity was the dominant factor responsible for the variations in BAF values in the rhizosphere soil (Figure 5a,b). Zeng et al. [47] indicated that the activities of soil enzymes can be stimulated by low concentrations of TMs. In this study, the relatively low concentrations of TMs in the rhizosphere soils irrigated with wastewater were in accordance with the China’s soil environmental quality standards (Table 2). In addition, the consistent distribution (Table 1 and Table 2) of catalase activity and TM concentrations implied that catalase activity might be stimulated by TMs in the rhizosphere soils irrigated with wastewater. Specifically, the results confirmed that soil Eh has a strong positive effect on the solubility of TMs in rhizosphere soil [48]. Therefore, increasing soil Eh could promote the solubility of TMs and further stimulate catalase activity [45], ultimately leading to a positive effect of soil Eh on catalase activity in TWIF and DWIF (Figure 5). Catalase activity relates to the metabolism activity of aerobic organisms and can be used as an indicator of soil fertility [36,49]. The increase in catalase activity reveals that the activity of aerobic organisms might be promoted, and the flux of active organic carbon might be increased in rhizosphere soil. Newly generated active OM constantly reacted with the TMs through ion exchange, adsorption, complexation, chelation, flocculation, and redox [50,51], thereby accelerating the transformation of TMs from NB to PB and PB to B (Figures S1 and S3), and eventually increasing the concentrations of PB and B in TWIF and DWIF. In addition, high concentrations of TMs contained in tanning wastewater may be another important factor for the increase in PB and B in TWIF (Table 2). The B part of TMs in TWIF and DWIF did not contribute to the variations in BAF values (Figure 6), possibly because its contribution to the variations in BAF values was mainly induced by increasing its flux rather than concentrations.

## 5. Conclusions

This study investigated the impact of long-term irrigation with wastewater from different sources on the uptake of TMs by plant roots in the soil rhizosphere microenvironment. Irrigation with tanning and domestic wastewater can led to an enhancement in the bioavailability of TMs in rhizosphere soils mainly by increasing active-soil organic carbon content, catalase activity and soil Eh to increase the concentrations of TMs in the PB part. Active organic carbon content and catalase activity had direct positive effects on BAF values, and soil Eh indirectly affected BAF values by influencing active organic carbon content and catalase activity in farmlands irrigated with tanning and domestic wastewater. Conversely, pharmaceutical wastewater irrigation reduced the bioavailability of TMs in rhizosphere soil by increasing soil antibiotic content to reduce the concentrations of TMs in the PB and B parts. Overall, our results indicate that the effects of wastewater irrigation on the bioavailability of TMs in rhizosphere soils were largely dependent on the source of wastewater. This work provides a new perspective on TM pollution in the soil rhizosphere microenvironment caused by diverse sources of wastewater irrigation. Further studies are required to clarify our findings and their universality in different soil types. In addition, given that tanning and domestic wastewater irrigation may enhance the risk of existing TM contamination, stringent regulations are needed for the irrigations using these wastewaters in the future, especially in developing countries.

## Figures and Tables

**Table 1:** Physicochemical characteristics and enzyme activities of the rhizosphere soils irrigated with groundwater and wastewater from different sources.

	Control	TWIF	PWIF	DWIF
TOC (g/kg)	12.19 ± 0.96	12.97 ± 4.88	17.40 ± 3.64 *	14.34 ± 1.48 *
DOC (g/kg)	1.08 ± 0.16	1.24 ± 0.10	1.95 ± 0.34 *	1.87 ± 0.19 *
KMnO_4_-C (g/kg)	2.03 ± 0.22	2.32 ± 0.23	4.27 ± 0.40 *	4.16 ± 0.56 *
Total antibiotics (µg/kg)	856.01 ± 108.33	910.31 ± 63.04	1534.69 ± 117.20 *	1007.66 ± 76.58
Soil pH	7.63 ± 0.39	7.52 ± 0.97	8.13 ± 0.68	6.85 ± 0.89
Clay (%)	35.00 ± 1.82	34.47 ± 3.54	39.50 ± 3.87 *	30.64 ± 2.80 *
Eh (mV)	72.48 ± 7.37	85.34 ± 8.90 *	63.96 ± 9.25 *	95.98 ± 8.48 *
CEC (cmol^+^/kg)	19.28 ± 3.58	24.04 ± 4.37 *	19.54 ± 2.01	16.58 ± 2.30
N (g/kg)	1.72 ± 0.34	1.54 ± 0.36	2.31 ± 0.32	2.39 ± 0.90
P (g/kg)	0.58 ± 0.06	0.59 ± 0.08	0.99 ± 0.11	0.82 ± 0.13
K (g/kg)	23.91 ± 4.27	19.34 ± 3.69	21.95 ± 7.03	27.22 ± 4.66
Ca (g/kg)	21.64 ± 8.90	18.74 ± 11.45	22.20 ± 8.91	18.98 ± 5.96
Mg (g/kg)	1.37 ± 1.25	0.79 ± 1.55	1.55 ± 1.11	1.15 ± 0.80
S (g/kg)	1.34 ± 0.34	1.28 ± 0.41	0.80 ± 0.27	0.91 ± 0.32
Fe (g/kg)	56.01 ± 28.30	73.60 ± 20.11 *	51.85 ± 25.12	52.23 ± 14.48
Mn (g/kg)	0.83 ± 0.21	1.01 ± 0.15	0.93 ± 0.11	0.98 ± 0.12
Catalase (mL/(20 mM KMnO_4_) h/g)	4.25 ± 1.54	11.27 ± 3.63 *	8.41 ± 1.92 *	9.86 ± 3.34 *
LiP (µmol/min/g)	1,43 ± 0.26	1.64 ± 0.35	1.09 ± 0.44 *	2.18 ± 0.65 *
MnP (µmol/min/g)	4.3 ± 0.62	4.41 ± 0.56	3.87 ± 0.82	4.46 ± 1.45
Lac (µmol/min/g)	1.91 ± 0.41	1.86 ± 0.21	1.52 ± 0.36 *	2.75 ± 0.44 *
Biomass (g/kg)	0.35 ± 0.05	0.26 ± 0.06 *	0.26 ± 0.08 *	0.44 ± 0.06 *

Notes: TWIF, PWIF, DWIF, and Control denote irrigation with tanning, pharmaceutical, domestic wastewater, and groundwater, respectively. Asterisks (*) indicate significant differences in physicochemical indices among rhizosphere soils irrigated with groundwater (control) and wastewater from different sources. TOC, total organic carbon. DOC, dissolved organic carbon. KMnO_4_-C, permanganate oxidizable carbon. Eh, soil redox potential. CEC, cation exchange capacity. Lip, lignin peroxidase. MnP, manganese peroxidase. Lac, laccase.

**Table 2:** Concentrations of trace metals (TMs) in rhizosphere soils irrigated with groundwater and different wastewater types (mg/kg dry wt).

	Control	TWIF	PWIF	DWIF	Background ^a^	Threshold ^b^
Cu	26.27 ± 2.33	40.06 ± 3.92 *	30.08 ± 3.86 *	30.53 ± 3.98 *	35	100
Cr	90.02 ± 10.47	189.61 ± 16.55 *	92.78 ± 6.61	100.88 ± 18.46 *	90	350
Cd	1.26 ± 0.16	1.71 ± 0.13 *	1.34 ± 0.16	1.24 ± 0.06	0.2	10
As	13.24 ± 1.08	13.52 ± 0.89	13.22 ± 1.30	11.63 ± 0.78 *	15	20
Pb	25.26 ± 1.18	43.94 ± 5.45 *	24.20 ± 0.86	26.32 ± 3.98	35	350
Ni	80.44 ± 8.72	105.43 ± 9.60 *	73.11 ± 5.83	78.99 ± 8.37	40	60

Notes: * Significant differences in the concentrations of TM among soils irrigated with groundwater and wastewater from different sources. ^a^ Background of Hebei province (MEPPRC, 1995) [37]. ^b^ The second grade of environmental quality standard of TMs for farmland soil in China (MEPPRC, 1995) [37].

## Supplementary Materials

The following are available online at https://www.mdpi.com/1660-4601/16/17/3218/s1: Table S1. Summary of indices reflecting physicochemical characteristics and soil enzyme activities of rhizosphere soils; Table S2. Physicochemical properties of groundwater and wastewater from different sources; Figure S1. Correlations between trace metal speciation and soil physicochemical characters in rhizosphere soils irrigated with tanning wastewater; Figure S2. Correlations between trace metal speciation and soil physicochemical characters in rhizosphere soils irrigated with pharmaceutical wastewater; Figure S3. Correlations between trace metal speciation and soil physicochemical characters in rhizosphere soils irrigated with domestic wastewater; Figure S4. Correlations among soil physicochemical characters in rhizosphere soils irrigated with tanning wastewater; Figure S5. Correlations among soil physicochemical characters in rhizosphere soils irrigated with domestic wastewater; Figure S6. Correlations among soil physicochemical characters in rhizosphere soils irrigated with pharmaceutical waste water.

## References

[1] Friedel J., Langer T., Siebe C., Stahr K. (2000). Effects of long-Term waste water irrigation on soil organic matter, soil microbial biomass and its activities in central Mexico. Biol. Fertil. Soils.

[2] Tan W., Wang G., Huang C., Gao R., Xi B., Zhu B. (2017). Physico-Chemical protection, rather than biochemical composition, governs the responses of soil organic carbon decomposition to nitrogen addition in a temperate agroecosystem. Sci. Total Environ..

[3] Hussain I., Raschid L., Hanjra M.A., Marikar F., Van Der Hoek W. (2002). Wastewater Use in Agriculture: Review of Impacts and Methodological Issues in Valuing Impacts.

[4] Maldonado V., Rubio Arias H., Quintana R., Saucedo R., Gutierrez M., Ortega J., Nevarez G. (2008). Heavy metal content in soils under different wastewater irrigation patterns in Chihuahua, Mexico. Int. J. Environ. Res. Public Health.

[5] Zhang Y., Dai J., Wang R., Zhang J. (2008). Effects of long-Term sewage irrigation on agricultural soil microbial structural and functional characterizations in Shandong, China. Eur. J. Soil Boil..

[6] Nan Z., Zhao C., Li J., Chen F., Sun W. (2002). Relations between soil properties and selected heavy metal concentrations in spring wheat (*Triticum aestivum L.*) grown in contaminated soils. Water Air Soil Pollut..

[7] Huang M., Zhou S., Sun B., Zhao Q. (2008). Heavy metals in wheat grain: Assessment of potential health risk for inhabitants in Kunshan, China. Sci. Total Environ..

[8] Fu Q., Hu H., Li J., Huang L., Yang H., Lv Y. (2009). Effects of soil polluted by cadmium and lead on production and quality of pepper (*Capsicum annuum L.*) and radish (*Raphanus sativus L.*). J. Food Agric. Environ..

[9] Fu Q., Zhao H., Li T., Hou R., Liu D., Ji Y., Zhou Z., Yang L. (2019). Effects of biochar addition on soil hydraulic properties before and after freezing-Thawing. Catena.

[10] Mekki A., Dhouib A., Sayadi S. (2006). Changes in microbial and soil properties following amendment with treated and untreated olive mill wastewater. Microbiol. Res..

[11] Cao C., Zhang Q., Ma Z.B., Wang X.M., Chen H., Wang J.J. (2018). Fractionation and mobility risks of heavy metals and metalloids in wastewater-Irrigated agricultural soils from greenhouses and fields in Gansu, China. Geoderma.

[12] Li B., Cao Y., Guan X., Li Y., Hao Z., Hu W., Chen L. (2019). Microbial assessments of soil with a 40-Year history of reclaimed wastewater irrigation. Sci. Total Environ..

[13] Hou R., Li T., Fu Q., Liu D., Li M., Zhou Z., Li L., Yan J. (2019). Characteristics of water–Heat variation and the transfer relationship in sandy loam under different conditions. Geoderma.

[14] Zhang C., Yu Z.G., Zeng G.M., Jiang M., Yang Z.Z., Cui F., Zhu M.Y., Shen L.Q., Hu L. (2014). Effects of sediment geochemical properties on heavy metal bioavailability. Environ. Int..

[15] Bose S., Bhattacharyya A. (2008). Heavy metal accumulation in wheat plant grown in soil amended with industrial sludge. Chemosphere.

[16] Massaquoi L.D., Ma H., Liu X.H., Han P.Y., Zuo S.M., Hua Z.X., Liu D.W. (2015). Heavy metal accumulation in soils, plants, and hair samples: An assessment of heavy metal exposure risks from the consumption of vegetables grown on soils previously irrigated with wastewater. Environ. Sci. Pollut. Res..

[17] Khan S., Cao Q., Zheng Y., Huang Y., Zhu Y. (2008). Health risks of heavy metals in contaminated soils and food crops irrigated with wastewater in Beijing, China. Environ. Pollut..

[18] Zhang J., Li H., Zhou Y., Dou L., Cai L., Mo L., You J. (2018). Bioavailability and soil-to-crop transfer of heavy metals in farmland soils: A case study in the Pearl River Delta, South China. Environ. Pollut..

[19] Bacon J.R., Davidson C.M. (2008). Is there a future for sequential chemical extraction?. Analyst.

[20] Tao S., Chen Y., Xu F., Cao J., Li B. (2003). Changes of copper speciation in maize rhizosphere soil. Environ. Pollut..

[21] Khoshgoftarmanesh A.H., Afyuni M., Norouzi M., Ghiasi S., Schulin R. (2018). Fractionation and bioavailability of zinc (Zn) in the rhizosphere of two wheat cultivars with different Zn deficiency tolerance. Geoderma.

[22] Ata R., Töre G.Y. (2019). Characterization and removal of antibiotic residues by NFC-Doped photocatalytic oxidation from domestic and industrial secondary treated wastewaters in Meric-Ergene Basin and reuse assessment for irrigation. J. Environ. Manag..

[23] Nguyen T.X.T., Amyot M., Labrecque M. (2017). Differential effects of plant root systems on nickel, copper and silver bioavailability in contaminated soil. Chemosphere.

[24] Chen Y., Shen Z., Li X. (2004). The use of vetiver grass (*Vetiveria zizanioides*) in the phytoremediation of soils contaminated with heavy metals. Appl. Geochem..

[25] Nayek S., Gupta S., Saha R. (2010). Metal accumulation and its effects in relation to biochemical response of vegetables irrigated with metal contaminated water and wastewater. J. Hazard. Mater..

[26] Tan W., Zhang Y., Xi B., He X., Gao R., Huang C., Zhang H., Li D., Zhao X., Li M. (2018). Discrepant responses of the electron transfer capacity of soil humic substances to irrigations with wastewaters from different sources. Sci. Total Environ..

[27] Wu D., Zhang C., Meng F. (2004). Economic loss evaluation of agricultural environmental pollution from wastewater irrigation in Hebei Province. Chin. J. Eco-Agric..

[28] Rhoades J. (1996). Salinity: Electrical conductivity and total dissolved solids. Methods Soil Anal. Part.

[29] Vieira F., Bayer C., Zanatta J., Dieckow J., Mielniczuk J., He Z. (2007). Carbon management index based on physical fractionation of soil organic matter in an Acrisol under long-term no-till cropping systems. Soil Tillage Res..

[30] Xie Y.F., Li X.W., Wang J.F., Christakos G., Hu M.G., An L.H., Li F.S. (2012). Spatial estimation of antibiotic residues in surface soils in a typical intensive vegetable cultivation area in China. Sci. Total Environ..

[31] Vance E.D., Brookes P.C., Jenkinson D.S. (1987). An extraction method for measuring soil microbial biomass C. Soil Boil. Biochem..

[32] Zhou Q., Wu Z., Cheng S., He F., Fu G. (2005). Enzymatic activities in constructed wetlands and di-n-butyl phthalate (DBP) biodegradation. Soil Biol. Biochem..

[33] Fujii K., Uemura M., Hayakawa C., Funakawa S., Kosaki T. (2013). Environmental control of lignin peroxidase, manganese peroxidase, and laccase activities in forest floor layers in humid Asia. Soil Biol. Biochem..

[34] Tessier A., Campbell P.G., Bisson M. (1979). Sequential extraction procedure for the speciation of particulate trace metals. Anal. Chem..

[35] Eisenhauer N., Bowker M.A., Grace J.B., Powell J.R. (2015). From patterns to causal understanding: Structural equation modeling (SEM) in soil ecology. Pedobiologia.

[36] Pascual J.A., Hernandez T., Garcia C., Ayuso M. (1998). Enzymatic activities in an arid soil amended with urban organic wastes: Laboratory experiment. Bioresour. Technol..

[37] Ministry of Environmental Protection of the Peoples’ Republic of China (MEPPRC) (1995). Environmental Quality Standard for Soils GB15618-1995.

[38] Zhao K., Liu X., Zhang W., Xu J., Wang F. (2011). Spatial dependence and bioavailability of metal fractions in paddy fields on metal concentrations in rice grain at a regional scale. J. Soils Sediments.

[39] Nourbakhsh F., Monreal C.M. (2004). Effects of soil properties and trace metals on urease activities of calcareous soils. Biol. Fertil. Soils.

[40] Adrover M., Farrús E., Moyà G., Vadell J. (2012). Chemical properties and biological activity in soils of Mallorca following twenty years of treated wastewater irrigation. J. Environ. Manag..

[41] Mancino C., Pepper I. (1992). Irrigation of turfgrass with secondary sewage effluent: Soil quality. Agron. J..

[42] Andersson D.I., Hughes D. (2014). Microbiological effects of sublethal levels of antibiotics. Nat. Rev. Microbiol..

[43] Tarchouna L.G., Merdy P., Raynaud M., Pfeifer H.R., Lucas Y. (2010). Effects of long-Term irrigation with treated wastewater. Part I: Evolution of soil physico-chemical properties. Appl. Geochem..

[44] Sauvé S., Hendershot W., Allen H.E. (2000). Solid-Solution partitioning of metals in contaminated soils: Dependence on pH, total metal burden, and organic matter. Environ. Sci. Technol..

[45] Shuzhuan W., Xiaorong W., Mingde H. (2016). Dynamics and availability of different pools of manganese in semiarid soils as affected by cropping system and fertilization. Pedosphere.

[46] Park J.H., Lamb D., Paneerselvam P., Choppala G., Bolan N., Chung J.W. (2011). Role of organic amendments on enhanced bioremediation of heavy metal (loid) contaminated soils. J. Hazard. Mater..

[47] Zeng L.S., Liao M., Chen C.L., Huang C.Y. (2007). Effects of lead contamination on soil enzymatic activities, microbial biomass, and rice physiological indices in soil–lead–rice (*Oryza sativa L.*) system. Ecotoxicol. Environ. Saf..

[48] Kashem M., Singh B. (2001). Metal availability in contaminated soils: I. Effects of floodingand organic matter on changes in Eh, pH and solubility of Cd, Ni and Zn. Nutr. Cycl. Agroecosyst..

[49] Guwy A., Martin S., Hawkes F., Hawkes D. (1999). Catalase activity measurements in suspended aerobic biomass and soil samples. Enzyme Microb. Technol..

[50] Weng L., Temminghoff E.J., Lofts S., Tipping E., Van Riemsdijk W.H. (2002). Complexation with dissolved organic matter and solubility control of heavy metals in a sandy soil. Environ. Sci. Technol..

[51] Gong C., Donahoe R.J. (1997). An experimental study of heavy metal attenuation and mobility in sandy loam soils. Appl. Geochem..

