# Supplementary material for: Biouptake Responses of Trace Metals to Long-Term Irrigation with Diverse Wastewater in the Wheat Rhizosphere Microenvironment
Source: Int J Environ Res Public Health. 2019 Sep 3;16(17):3218. doi: 10.3390/ijerph16173218 (PMC6747335; doi:10.3390/ijerph16173218)
Supplement: Supplementary file 1 [file ijerph-16-03218-s001.pdf]

-Supplementary Material-

Table S1. Integrated wastewater discharge standard of China (mg/L).

	Scope of Application	Primary Standard	Secondary Standard	Third Standard
pH	All discharge units		6–9	
COD	Sugar beet, dye, scouring, organophosphorus pesticide industry	100	200	1000
	Monosodium glutamate, alcohol, pharmaceutical raw materials, pharmaceutical, Tanning industry	100	300	1000
	Petrochemical industry	60	120	500
	Municipal sewage treatment plant	60	120	-
	Other discharge units	100	150	500
BOD_5	Sugar cane sugar, ramie degumming, wet fiberboard, dye, scouring industry	20	60	600
	Beet sugar, alcohol, monosodium glutamate, tanning, chemical fiber pulp industry	20	100	6000
	Municipal sewage treatment plant	20	30	-
	Other discharge units	20	30	300
SS	Mining, beneficiation, coal preparation industry	70	300	-
	Vein gold dressing industry	70	400	-
	Placer gold ore dressing industry	70	800	-
	Municipal sewage treatment plant	20	30	-
	Other discharge units	70	150	400
NH_3-N	Pharmaceutical raw materials, dyes, petrochemical industry	15	50	-
	Other discharge units	15	25	-
TP	All discharge units	1	3	5
Methanal	All discharge units	1	2	5
Nitrobenzene	All discharge units	2	3	5
Aniline	All discharge units	1	2	5
Mn	Synthetic fatty acid industry	2	5	5
	Other discharge units	2	2	5
Cu	All discharge units	0.5	1	2
Zn	All discharge units	2	5	5
Hg	All discharge units		0.05	
Cd	All discharge units		0.1	
Cr	All discharge units		1.5	
Cr (VI)	All discharge units		0.5	
As	All discharge units		0.5	
Pb	All discharge units		1.0	
Ni	All discharge units		1.0	
Ag	All discharge units		0.5	

Table S2. Physicochemical properties of groundwater and wastewater from different sources.

	Control	PWIF	TWIF	DWIF
pH	7.16 ± 0.26	7.75 ± 0.34	7.22 ± 0.19	7.87 ± 0.23
EC (mS/cm)	1.27 ± 0.33	1.11 ± 0.13	2.75 ± 0.25	2.09 ± 0.54
DOC (mg/L)	1.56 ± 0.47	35.7 ± 3.55	14.7 ± 0.80	32.4 ± 4.22
BOD5 (mg/O^2/L)	2.80 ± 0.67	67.3 ± 9.78	25.2 ± 6.74	61.8 ± 12.4
COD (mg/O^2/L)	13.4 ± 2.10	141 ± 20.9	63.2 ± 4.18	159 ± 31.8
Total N (mg/L)	0.50 ± 0.08	5.5 ± 0.87	2.15 ± 0.35	6.3 ± 1.34
Total P (mg/L)	0.12 ± 0.03	0.72 ± 0.08	0.14 ± 0.02	1.04 ± 0.13
Total antibiotics (μg/L)	ND	64.9 ± 16.4	ND	1.85 ± 0.41
K (mg/L)	3.11 ± 0.93	85.6 ± 19.6	108 ± 27.1	124 ± 9.85
Cr (μg/L)	2.12 ± 0.54	3.09 ± 0.17	26.6 ± 3.84	4.12 ± 0.83
Cd (μg/L)	ND	ND	21.4 ± 2.29	0.04 ± 0.01
As (μg/L)	ND	ND	2.78 ± 0.82	1.19 ± 0.19
Pb (μg/L)	0.09 ± 0.02	0.11 ± 0.02	45.6 ± 8.53	5.63 ± 1.31
Ca (mg/L)	25.4 ± 4.15	27.6 ± 8.06	34.2 ± 5.82	32.1 ± 2.74

Mg (mg/L)	23. 8 ± 5.52	24.8 ± 3.74	27.6 ± 2.69	28.3 ± 3.57
Ni (μg/L)	0.72 ± 0.06	1.13 ± 0.11	30.7 ± 5.89	14.5 ± 2.80
Fe (μg/L)	41.2 ± 2.66	38.6 ± 3.56	168 ± 20.2	65.3 ± 11.4
Mn (μg/L)	1.68 ± 0.39	1.79 ± 0.50	194 ± 15.3	41.5 ± 8.43
Cu (μg/L)	2.34 ± 0.61	2.06 ± 0.53	57.8 ± 6.99	2.68 ± 0.45
Zn (μg/L)	1.82 ± 0.25	2.39 ± 0.39	63.7 ± 8.30	20.4 ± 3.14

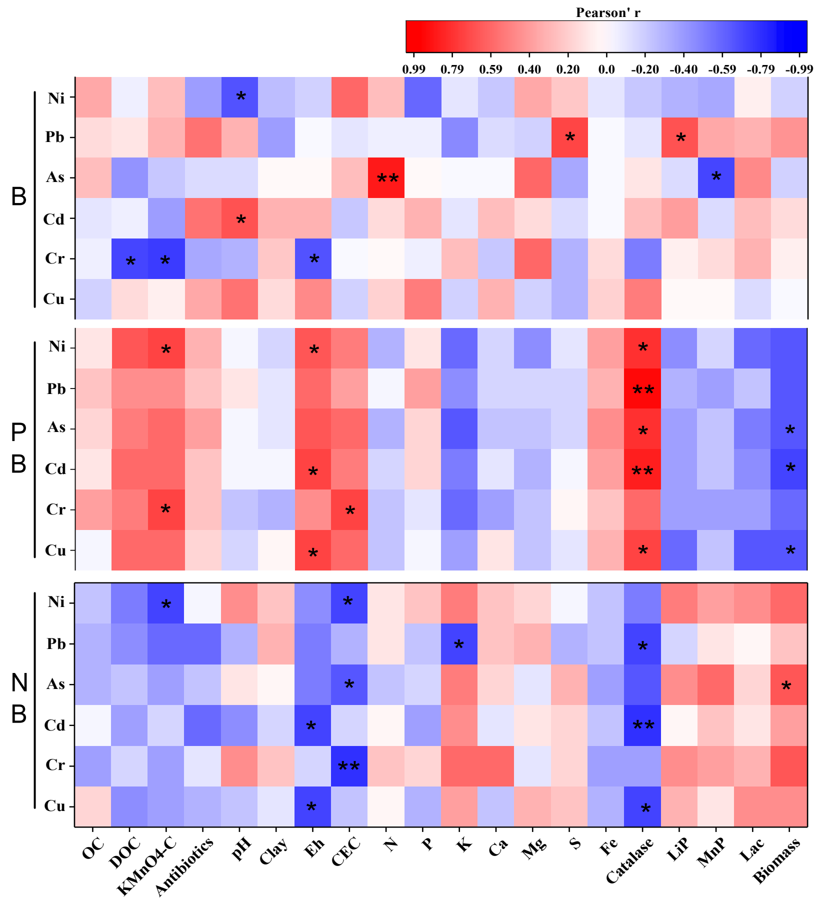

Figure S1. Correlations between heavy metal speciation (based on bioavailability) and soil physicochemical characters in rhizosphere soils irrigated with tanning wastewater. B: bioavailable part; PB: potentially bioavailable part; NB: non-bioavailable part.

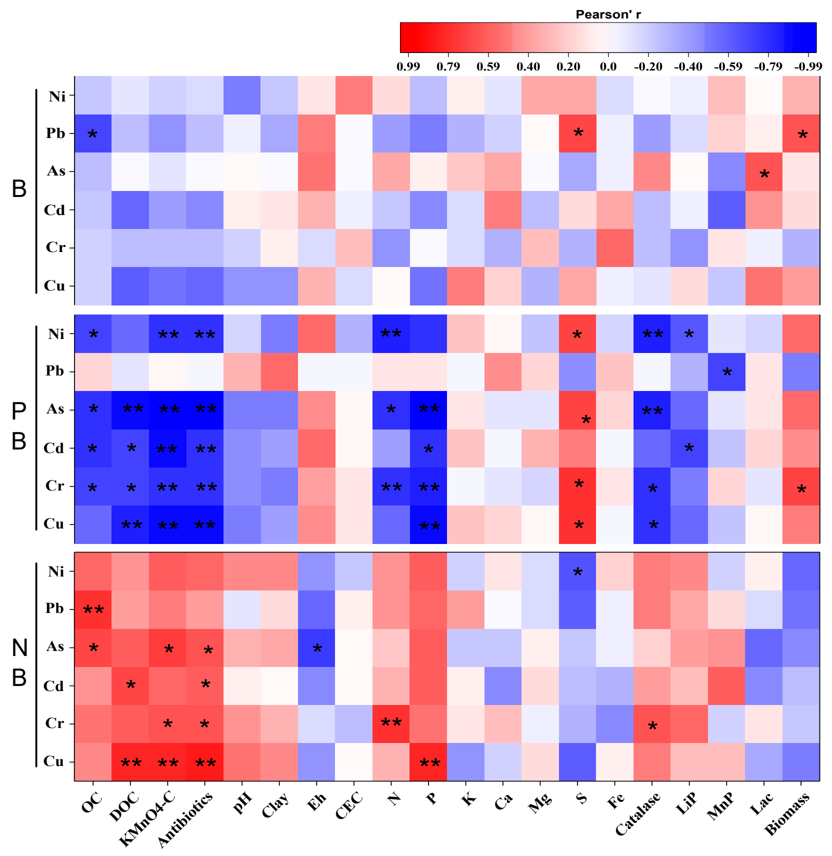

Figure S2. Correlations between heavy metal speciation (based on bioavailability) and soil physicochemical characters in rhizosphere soils irrigated with pharmaceutical wastewater. B: bioavailable part; PB: potentially bioavailable part; NB: non-bioavailable part.

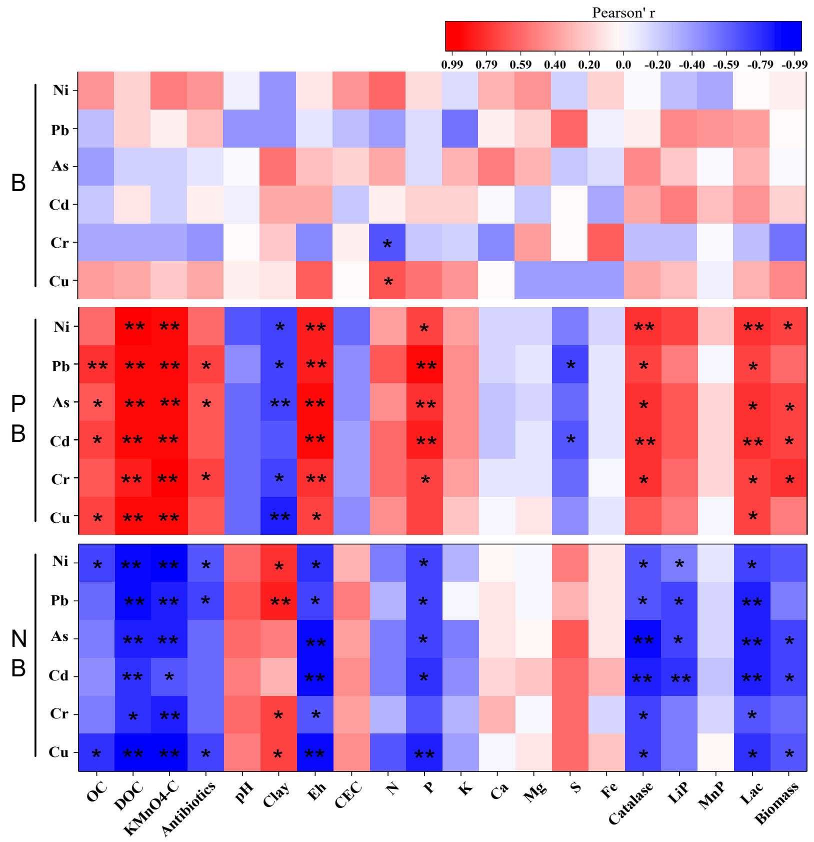

Figure S3. Correlations between heavy metal speciation (based on bioavailability) and soil physicochemical characters in rhizosphere soils irrigated with domestic wastewater. B: bioavailable part; PB: potentially bioavailable part; NB: non-bioavailable part.

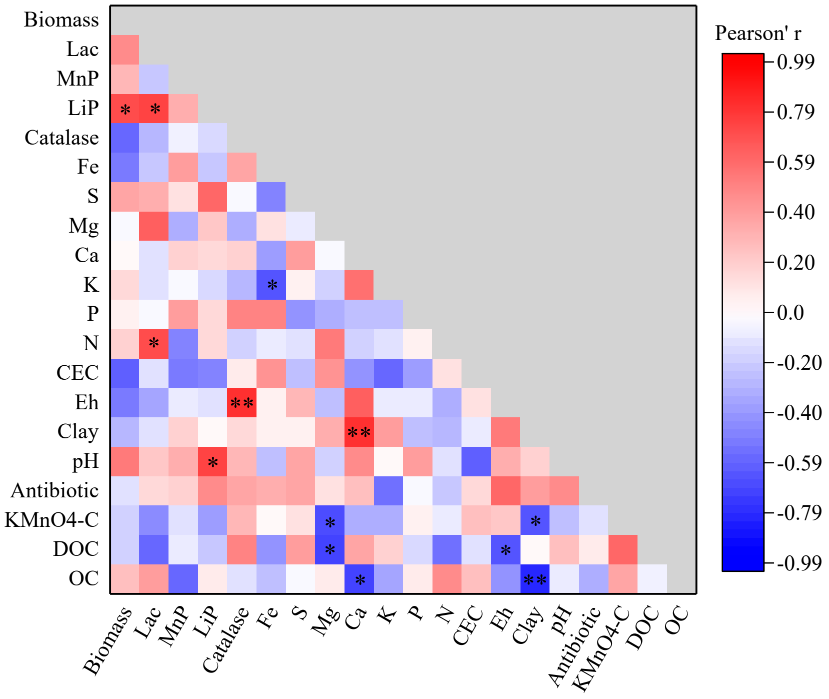

Figure S4. Correlations among soil physicochemical characters in rhizosphere soils irrigated with tanning wastewater.

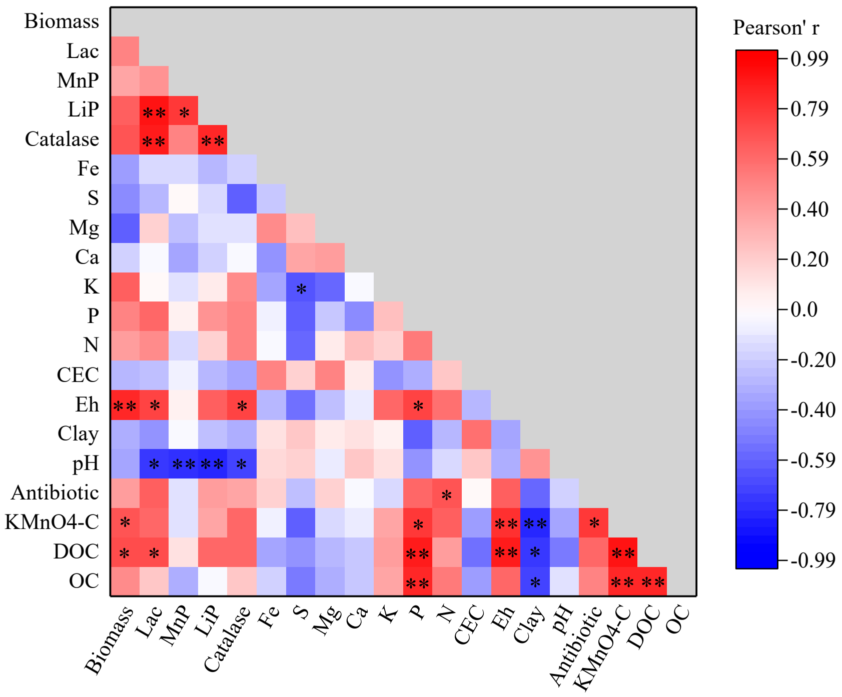

Figure S5. Correlations among soil physicochemical characters in rhizosphere soils irrigated with domestic wastewater.

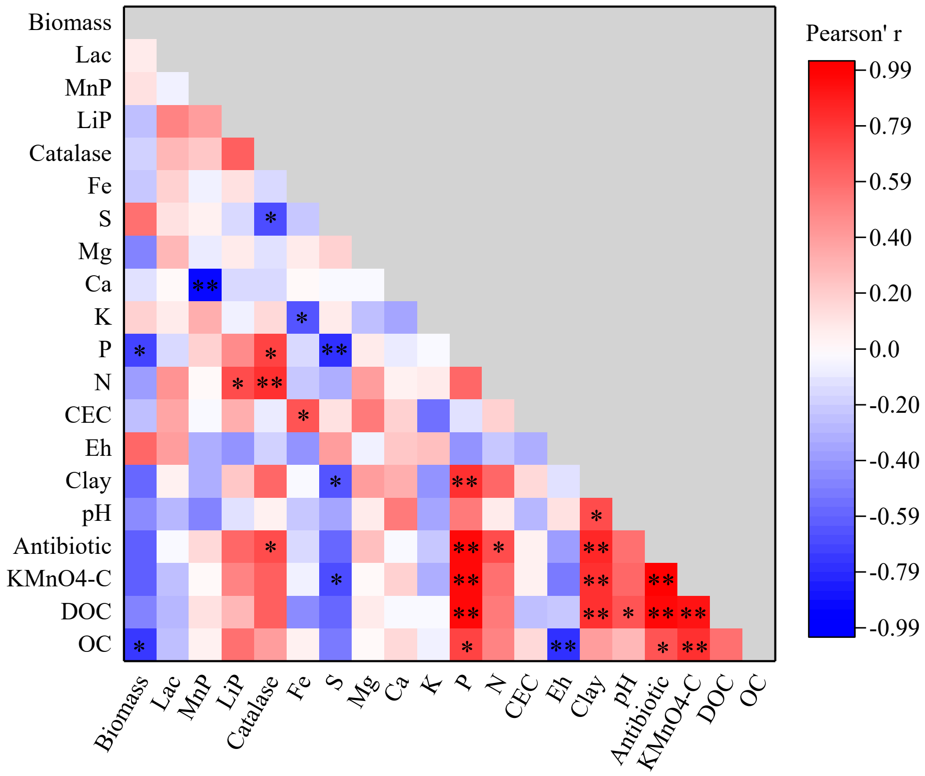

Figure S6. Correlations among soil physicochemical characters in rhizosphere soils irrigated with pharmaceutical waste water.
